# Correction to inhibition of microRNA let‐7b expression by KDM2B promotes cancer progression by targeting EZH2 in ovarian cancer

**DOI:** 10.1111/cas.16291

**Published:** 2024-07-23

Yan Kuang, Hong Xu, Fangfang Lu, Jiahua Meng, Yeye Yi, Huilan Yang, Hairui Hou, Hao Wei, Shanheng Su. Inhibition of microRNA let‐7b expression by KDM2B promotes cancer progression by targeting EZH2 in ovarian cancer. Cancer Science. 2021;112:231–242.

There were errors in Figure 4. The migration images of SKOV3 blank control were misselected. The correct Figure 4 is shown below:

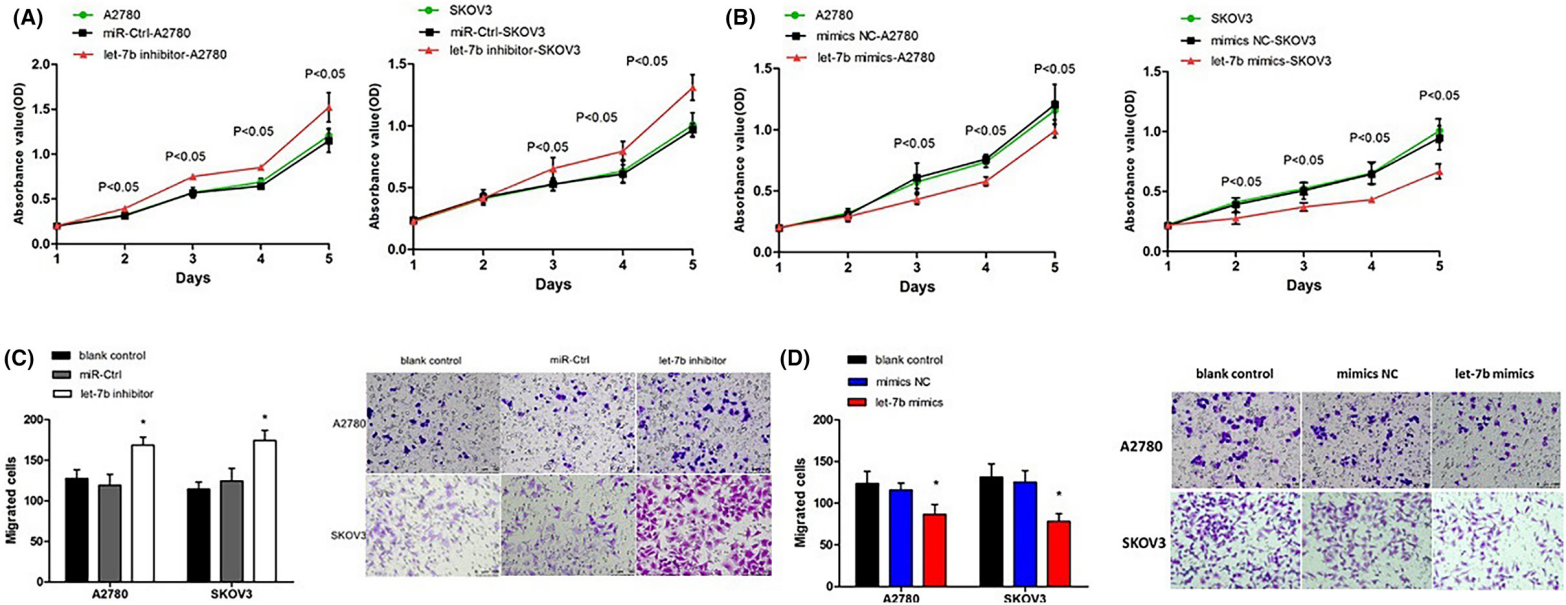

There was an error in Figure 5E. The migration image of A2780 LV‐KDM2B was misused. The correct Figure 5 is shown below:

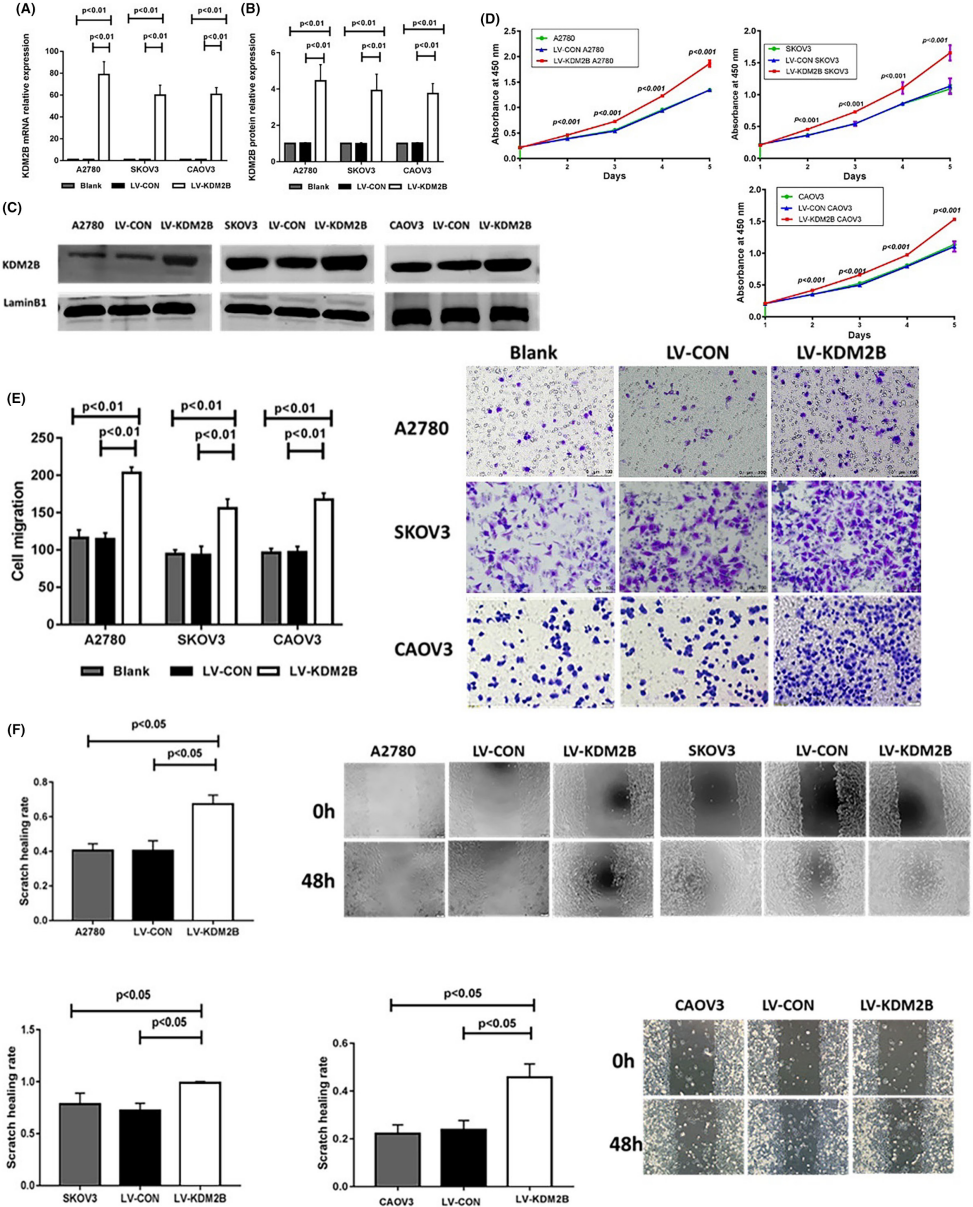

There were errors in Figure 6B. The migration images of SKOV3 LV‐KDM2B + miR‐Ctrl and A2780 LV‐KDM2B were misused. The correct Figure 6 is shown below:

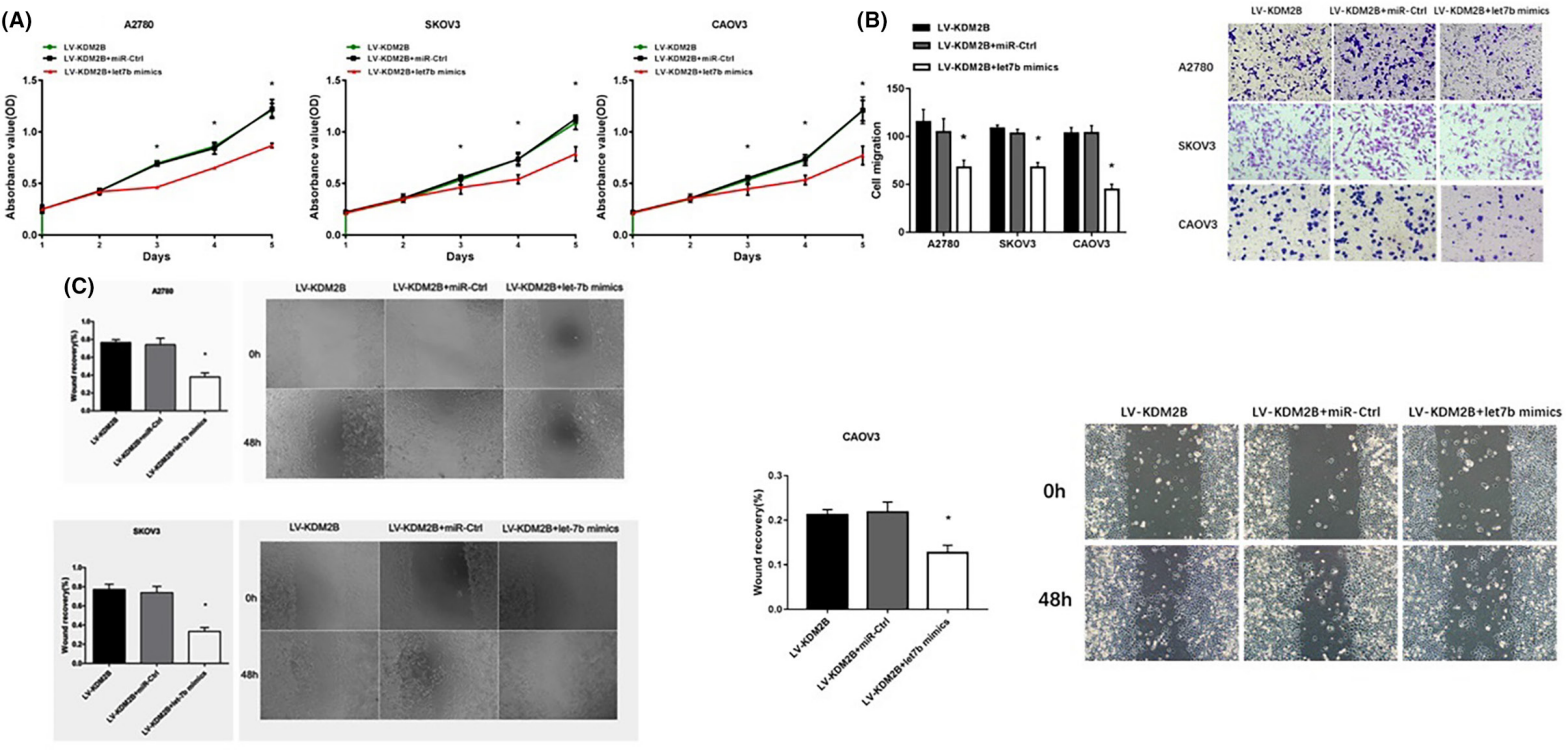

We apologize for these errors.

